# Safely Launched and in Port

**Published:** 1916-04-08

**Authors:** 


### THE MATRONS' AND SISTERS' DEPARTMENT.

### SAFELY LAUNCHED AND IN PORT.

THE good ship *College of Nursing* has been safely launched. It was registered on Monday, March 27, 1916, not as a joint-stock company with shares, but as a company limited by guarantee. The objections at first urged against this form of legal incorporation have been thus rendered non-effective and meaningless in fact. The next stage was to find, pass, and appoint the managers and officers. This important stage has proved so successful that it demonstrates how completely Mr. Stanley's proposal has won its way to acceptance throughout the nursing world of Great Britain. Is there any one of us, however wide our experience or complete our knowledge, that could have prepared a list of the real leaders of British nursing more representative, and on the whole more reasonably complete, than the first Council of the College of Nursing, which includes the following members of the nursing profession: Miss Haughton (Guy's Hospital), Miss Lloyd Still (St. Thomas's Hospital), Miss McIntosh (St. Bartholomew's Hospital), Miss Cox-Davies, R.R.C. (Royal Free Hospital), Miss Ray, R.R.C. (King's College), Miss Barton (Chelsea Infirmary), Miss Mowat (Whitechapel Infirmary), Miss Amy Hughes (Q.V.J.N.I.), Miss Swift, R.R.C. (Matron-in-Chief, Joint War Committee), Miss Sparshott, R.R.C. (Manchester Royal Infirmary), Miss Vincent, R.R.C. (Leicester Royal Infirmary), Miss Baillie, R.R.C. (Bristol Royal Infirmary), Miss Musson, R.R.C. (Birmingham General Hospital), Miss Yapp (Poor-Law Hospital, Ashton-under-Lyne), Miss Gill, R.R.C. (Edinburgh Royal Infirmary), Miss Melrose, R.R.C. (Glasgow Royal Infirmary)? The list as arranged presents the names in the order of (1) the metropolitan training schools; (2) metropolitan Poor-Law infirmaries; (3) provincial training schools and hospitals; (4) training schools in Scotland; (5) general nursing and the Joint War Committee. It still remains to co-opt representatives of Ireland and Wales, of the practising nurse, and possibly some other branch of nursing. It would be invidious to analyse the names in detail, but we may properly congratulate Mr. Stanley upon the remarkably representative Council which it has been possible for him and his advisers to secure at the outset.

The Hon. Arthur Stanley will, of course, be the first chairman of the Council, and a source of strength and knowledge is the fact that Sir Cooper Perry, with great self-denial and a fine public spirit, has consented to become the first honorary secretary of the College. With them are associated Mr. Comyns Berkeley, F.R.C.P., treasurer of the Royal British Nurses' Association; Professor Glaister, M.D., Regius Professor of Forensic Medicine and Public Health, University of Glasgow; Dr. Jane Walker, Dr. Turney, M.A., and Mr. James Cantlie. It will be seen how representative and inclusive the Council of the College has become within one week of its incorporation. The two first steps in the history of the College have so been taken with remarkable judgment and complete success.

This issue of THE HOSPITAL will reach our readers before the great meeting is held at St. Thomas's Hospital, on Friday, April 7, to which Mr. Stanley has invited the attendance of the most knowledgeable representatives of the training schools and principal hospitals throughout the country. This meeting should result in the formation of a widely representative Consultative Board with full powers to proceed at once to the formation of small sub-committees, the duty of which will be to determine many questions of initial importance, including curricula, examinations, and many other matters. Friday's meeting should give a tremendous impetus not only to the Council, the consultative body, and the numerous committees, but to the College's resources and influence. It should secure the inspiration of all shades of opinion throughout the nursing profession whose main aim really is to bring to it and to British nurses adequate recognition and a status resting upon approved standards. The absence of all this in the past has led to much of the chaos and most of the disappointments that have too long beset the progressive development of nurse education, training, and service throughout the United Kingdom.

There is every prospect as we write that the Council and all connected with the College of Nursing will be in a position to commence their work with every encouragement and co-operation they could wish for. Some of the ground has already been well prepared by the abler leaders of nursing in these islands. Great hospitals and schools fortunate in the possession of a matron of high administrative ability already have an accurate, up-to-date, and perfected register of the nurses trained by the school for which she is responsible. We have, therefore, great hope that it will be found possible forthwith to make a register which will immediately embrace upwards of 20,000 nurses of the highest proficiency and training. Few more interesting—we had almost said fascinating—responsibilities could be undertaken by the leaders of nursing in this country, than the equipment and organisation of the College of Nursing, the mere formulation of which has accomplished the miracle of uniting all the nurse-training schools, and practically everybody, who is anybody, at present engaged in the active practice of the profession of nursing.

